# Trace amines inhibit insect odorant receptor function through antagonism of the co-receptor subunit

**DOI:** 10.12688/f1000research.3825.1

**Published:** 2014-04-03

**Authors:** Sisi Chen, Charles W. Luetje

**Affiliations:** 1 Department of Molecular and Cellular Pharmacology, University of Miami Miller School of Medicine, Miami, FL, 33101, USA

## Abstract

Many insect behaviors are driven by olfaction, making insect olfactory receptors (ORs) appealing targets for insect control.  Insect ORs are odorant-gated ion channels, with each receptor thought to be composed of a representative from a large, variable family of odorant binding subunits and a highly conserved co-receptor subunit (Orco), assembled in an unknown stoichiometry.  Synthetic Orco directed agonists and antagonists have recently been identified.  Several Orco antagonists have been shown to act via an allosteric mechanism to inhibit OR activation by odorants.  The high degree of conservation of Orco across insect species results in Orco antagonists having broad activity at ORs from a variety of insect species and suggests that the binding site for Orco ligands may serve as a modulatory site for compounds endogenous to insects or may be a target of exogenous compounds, such as those produced by plants.  To test this idea, we screened a series of biogenic and trace amines, identifying several as Orco antagonists.  Of particular interest were tryptamine, a plant-produced amine, and tyramine, an amine endogenous to the insect nervous system.  Tryptamine was found to be a potent antagonist of Orco, able to block Orco activation by an Orco agonist and to allosterically inhibit activation of ORs by odorants.  Tyramine had effects similar to those of tryptamine, but was less potent.  Importantly, both tryptamine and tyramine displayed broad activity, inhibiting odorant activation of ORs of species from three different insect orders (Diptera, Lepidoptera and Coleoptera), as well as odorant activation of six diverse ORs from a single species (the human malaria vector mosquito, *Anopheles gambiae*).  Our results suggest that endogenous and exogenous natural compounds serve as Orco ligands modulating insect olfaction and that Orco can be an important target for the development of novel insect repellants.

## Introduction

Insects have positive and negative impacts on humans, in terms of health, economy, and food stores. Insects pollinate plants to increase global food production, with 35% of global production of crops depending on animal pollinators ^1, 2^. Insects also cause significant destruction of crops and food stores ^3– 5^. Insects can also transmit fatal diseases such as dengue fever ^6^, malaria ^7^, yellow fever and epidemic typhus ^8^. Insects use olfaction to sense their surroundings and to guide important activities, including feeding, mating and oviposition. This makes the insect olfactory system receptors an attractive target for the chemical control of deleterious insect species.

Insects use odorant receptors (ORs) to recognize and distinguish a diverse range of odorants ^9, 10^. Each OR is composed of two functionally essential parts: a highly conserved co-receptor subunit (Orco) and one of a large number of variable odorant-binding (or “tuning”) subunits ^11– 17^. These subunits associate in an unknown stoichiometry to form an odorant-gated ion channel ^18, 19^. ORs have also been proposed to initiate, or be modified by, second messenger cascades ^13, 18^. While the odorant-binding subunit is responsible for interacting with odorants ^9, 20, 21^, both the odorant-binding subunits and Orco are involved in forming the ion channel pore ^21, 22^. Insect ORs are not related to the receptors and channels of humans and other tetrapods ^15^, suggesting that control of detrimental insect activity may be possible through the development of insect OR selective compounds. A current approach to developing these compounds is to identify the particular odorant binding subunits that recognize behaviorally important odorants ^10, 23– 26^ and then conduct large scale ligand screens ^27, 28^, but high diversity among the odorant binding subunit repertoires of different species makes this approach exceptionally labor intensive ^29, 30^.

The recent identification of the synthetic compound VUAA1 as a novel OR agonist that acts directly on Orco ^27^, suggests that manipulation of insect behavior might be achieved by targeting Orco. Based on the VUAA1 structure, several additional synthetic Orco agonists and a larger, more diverse series of synthetic Orco antagonists have been identified ^31– 33^. Importantly, several of these Orco antagonists were shown to inhibit odorant activation of ORs through a non-competitive mechanism ^31– 33^. These findings suggest that Orco antagonists might be useful in altering insect behavior.

Orco subunits are highly conserved across insect species, suggesting that Orco serves an essential function common to all insect ORs ^15, 34^. This high conservation underlies observations that Orco subunits from different species are functionally interchangeable; an Orco subunit from one species can form functional ORs with an odorant-binding subunit from a different species ^21, 22^. As the “pharmacology” of synthetic Orco agonists and antagonists has expanded, it has also become clear that Orco subunits from disparate insect species have very similar sensitivities to known Orco ligands ^27, 31– 33, 35^. This suggested to us that the binding site for Orco ligands may serve as a modulatory site for compounds endogenous to the insects or may be a target of exogenous compounds, such as those generated by plants. Insects use a variety of amines as neurotransmitters and neuromodulators ^36– 39^. Plants also generate a variety of amines that may play a role in resistance to insect herbivores ^40– 42^. For these reasons, we screened a panel of biogenic and trace amines for agonist and antagonist activity at insect Orco subunits. We found tryptamine to be a potent Orco antagonist with broad activity at Orco subunits from different species. Tyramine and phenethylamine also function as Orco antagonists, but were substantially less potent than tryptamine. Importantly, we found that tryptamine, acting through Orco, could inhibit odorant activation of a wide range of ORs from a variety of insect species. Our findings suggest a role for Orco as a modulatory site common to all insect ORs and support the development of Orco-directed compounds that can be used to manipulate insect behavior.

## Methods

### Materials

**Figure 1.**
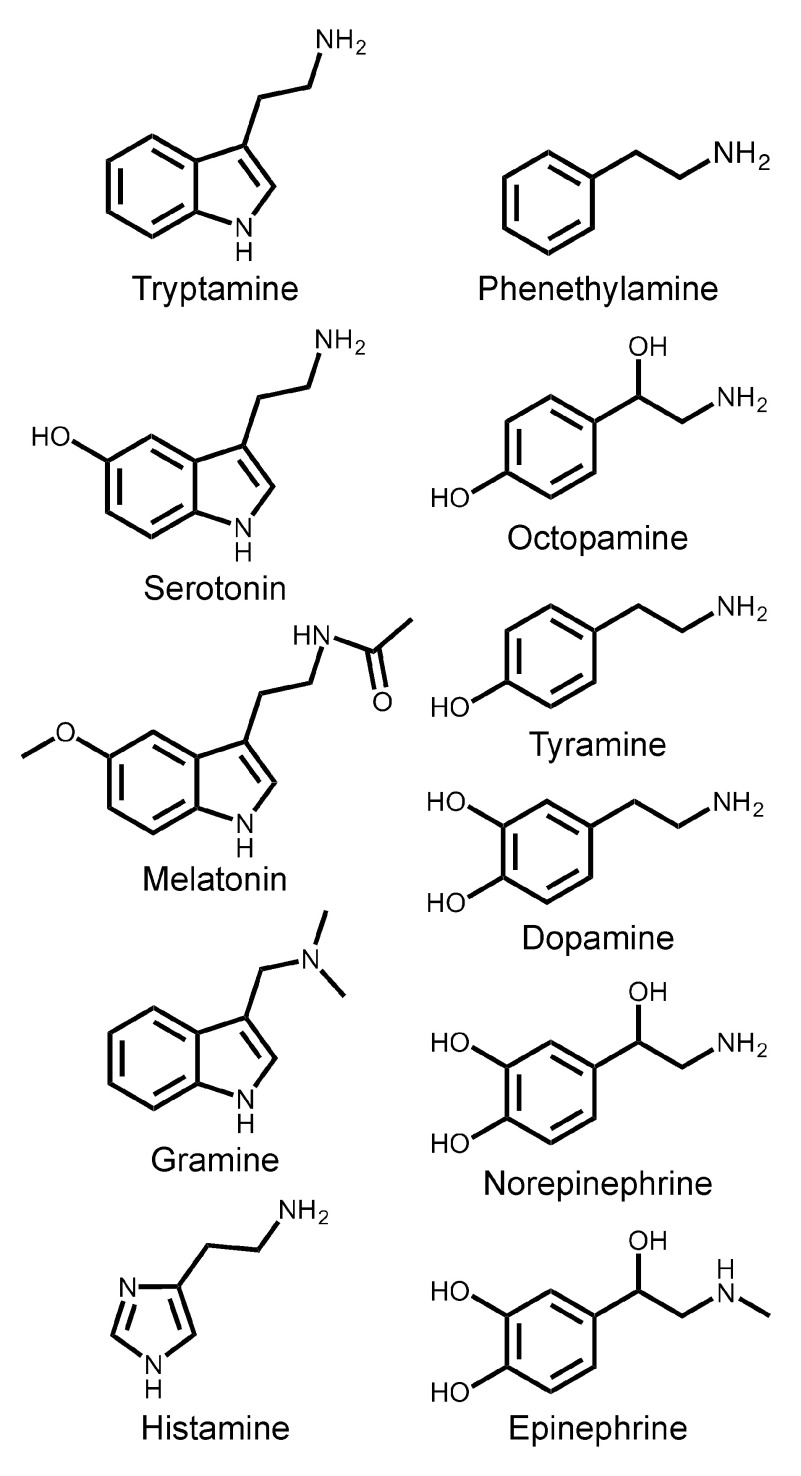
Structures of amines tested in this study.

*Xenopus laevis* frogs were purchased from Nasco (Fort Atkinson, WI). The care and use of *Xenopus laevis* frogs in this study were approved by the University of Miami Animal Research Committee (Animal Welfare Assurance #A-3224-01, Protocol #13-056) and meet the guidelines of the US National Institutes of Health. All experimentation was conducted on cultured oocytes after surgical removal from the frogs (see below). The amines screened in this study ( Figure 1), odorants (L-fenchone, acetophenone, geranyl acetate, 6-methyl-5-hepten-2-one, 2-nonanone and eugenol), OLC12 and other chemicals were from Sigma-Aldrich. Cqui\Orco (from *Culex quinquefasciatus*), Onub\Or6, Onub\Orco (from *Ostrinia nubilalis*), Mcar\Or5 and Mcar\Orco (from *Megacyllene caryae*) were cloned and inserted into the pGEMHE vector ^43^ as previously described ^23, 24, 44, 45^. Dmel\Or35a and Dmel\Orco (from *Drosophila melanogaster*) were generously provided by J. Carlson and L. Vosshall, respectively. Agam\Or27, Agam\Or28, Agam\Or31, Agam\Or39, Agam\Or48, Agam\Or65 and Agam\Orco (from *Anopheles gambiae*) were generously provided by L. Zweibel.

### Expression of insect ORs in *Xenopus* oocytes

Mature *Xenopus laevis* frogs were anesthetized by submersion in 0.1% 3-aminobenzoic acid ethyl ester. Depth of anesthesia was judged by loss of nasal flare and swallow reflexes. Oocytes were surgically removed. The incision was treated with gentamicin sulfate (two subcutaneous injections of 0.1 mL 10 mg/mL gentamycin at the surgical site) and sutured. Immediately following surgery (and before recovery from anesthesia), as an analgesia agent, one subcutaneous injection of Meloxicam solution (0.1 mg/mL) (0.1 mg/kg body weight) was administered to the dorsal lymph sac of the frogs. The frogs were allowed to recover from surgery in a humid chamber before being placed back in the holding tank. Surgeries were performed on individual frogs no more often than once every 3 months. Following the fourth surgery, frogs were anesthetized as described above and then pithed.

Follicle cells were removed by treatment with collagenase B (Boehringer Mannheim) for 2 hours at room temperature. Capped cRNA encoding each OR subunit was generated using mMessage mMachine kits (Ambion). For heteromeric ORs, 25 ng of cRNA encoding each OR subunit was injected into Stage V-VI *Xenopus* oocytes. For expression of Orco homomers, 50 ng of cRNA was injected. Oocytes were incubated at 18°C in Barth's saline (in mM: 88 NaCl, 1 KCl, 2.4 NaHCO _3_, 0.3 CaNO _3_, 0.41 CaCl _2_, 0.82 MgSO _4_, 15 HEPES, pH 7.6, and 150µg/ml ceftazidime) for 2–5 days prior to electrophysiological recording.

### Electrophysiology and data capture

Odorant and Orco ligand induced currents were recorded under two-electrode voltage clamp, using an automated parallel electrophysiology system (OpusExpress 6000A, Molecular Devices). Oocytes were perfused with ND96 (in mM: 96 NaCl, 2 KCl, 1 CaCl _2_, 1 MgCl _2_, 5 HEPES, pH 7.5). Orco ligands were prepared as 50 or 100 mM stock solutions in DMSO and then diluted into ND96 on the day of the experiment. Odorants were prepared as 100 mM stock solutions in DMSO and then diluted into ND96. Unless otherwise noted, applications were for 60 sec at a flow rate of 1.0 ml/min, with extensive washing in ND96 at 4.6 ml/min between applications. Micropipettes were filled with 3 M KCl and had resistances of 0.2–2.0 MΩ. The holding potential was -70 mV. Current responses, filtered (4-pole, Bessel, low pass) at 20 Hz (-3 db) and sampled at 100 Hz, were captured and stored using OpusXpress 1.1 software (Molecular Devices).

### Experimental protocols and data analysis

**Figure 2.**
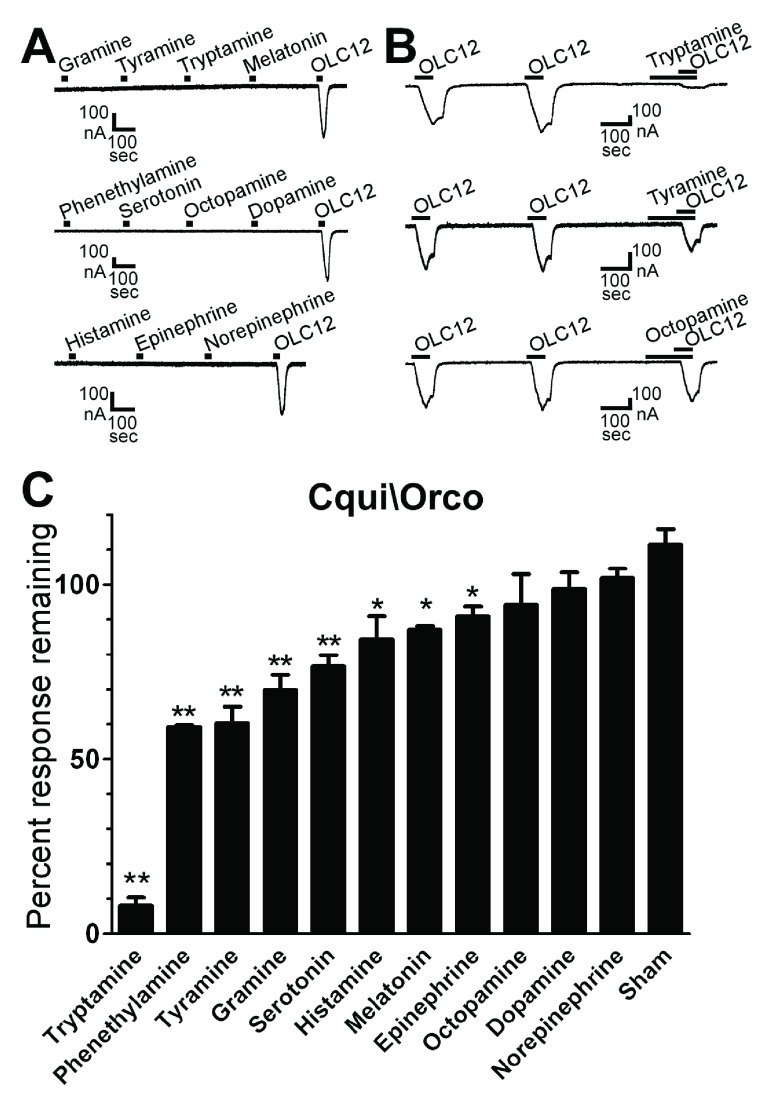
Tryptamine and several other amines are antagonists of Cqui\Orco. **A**) The tested amines do not display Orco agonist activity. Oocytes expressing Cqui\Orco were challenged with 30 sec applications of 100µM gramine, tyramine, tryptamine and melatonin (top trace), phenethylamine, serotonin, octopamine and dopamine (middle trace), or histamine, epinephrine and norepinephrine (bottom trace), with 5 min washes between applications. 30µM OLC12 (Orco agonist) was applied at the end of each trace. **B**) Tryptamine and tyramine are antagonists of Cqui\Orco. Oocytes expressing Cqui\Orco were exposed to 60 sec applications of 30µM OLC12 with 4 min washes between applications. 100µM tryptamine (top trace), tyramine (middle trace), or octopamine (bottom trace) were applied and incubated for 90 sec preceding the third application of OLC12 and then co-applied during the OLC12 application. **C**) Screen of 11 amines for Orco antagonism. Responses of Cqui\Orco to 30µM OLC12 (~EC _5_) in the presence of 100µM of each compound are presented as a percentage of the average of two preceding responses to OLC12 alone (mean ± SEM, n = 3-10). Statistical significance was assessed by one-way ANOVA, followed by Dunnett's post-test comparing to sham treated oocytes (*p<0.01; **p<0.001).

**Figure 3.**
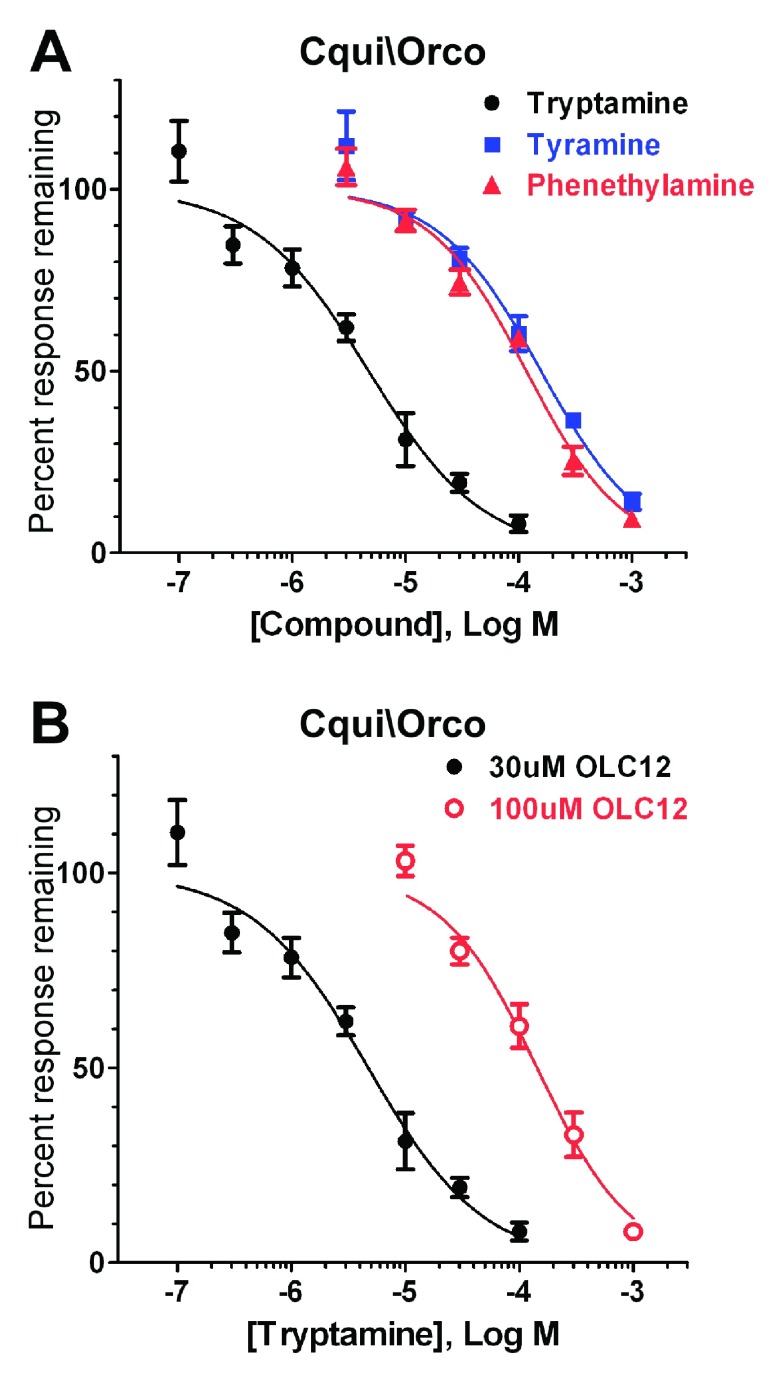
Trace amine antagonists of Cqui\Orco. **A**) Concentration-inhibition curves for tryptamine, tyramine and phenethylamine inhibition of Cqui\Orco activated by 30µM OLC12. **B**) Altering the concentration of Orco agonist (OLC12) shifts the tryptamine inhibition curve. The IC _50_ for tryptamine inhibition of Cqui\Orco activation by 30µM OLC12 (4.7 ± 0.7µM, n = 5) is significantly different (p<0.0001, F-test) from the IC _50_ for tryptamine inhibition of Cqui\Orco activation by 100µM OLC12 (143 ± 18µM, n = 6).

**Figure 4.**
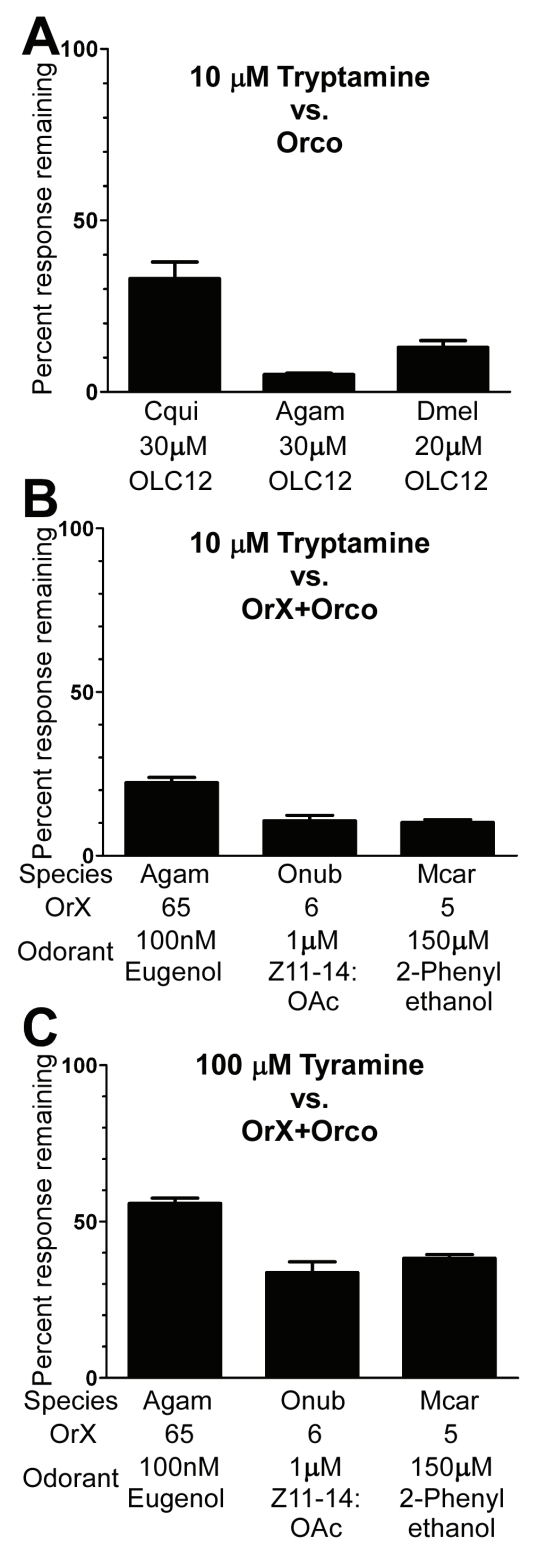
Tryptamine and tyramine inhibit odorant activation of ORs from different insect species. **A**) Oocytes expressing Orco from each of three different species were activated by the indicated concentration of OLC12. For Cqui\Orco from *Cx. quinquefasciatus*, 30µM is the ~EC _5_; for Agam\Orco from *An. gambiae*, 30µM is the ~EC _3_; for Dmel\Orco from *D. melanogaster*, 20µM is the ~EC _10_. Current responses in the presence of 10µM tryptamine were compared to the average of two preceding responses to OLC12 and are presented as mean ± SEM (n = 4-9). **B**– **C**) Tryptamine and tyramine inhibit odorant activation of heteromeric ORs from different insect species. Oocytes expressing an OR from *An. gambiae* (Agam\Orco+Agam\Or65) were activated by 100nM eugenol, oocytes expressing an OR from *O. nubilalis* (Onub\Orco+Onub\Or6) were activated by 1µM Z11-14:OAc, oocytes expressing an OR from *M. caryae* (Mcar\Orco+Mcar\Or5) were activated by 150µM 2-phenylethanol. Current responses in the presence of 10µM tryptamine ( **B**) or 100µM tyramine ( **C**) were compared to the preceding response to odorant alone and are presented as mean ± SEM (n = 3).

**Figure 5.**
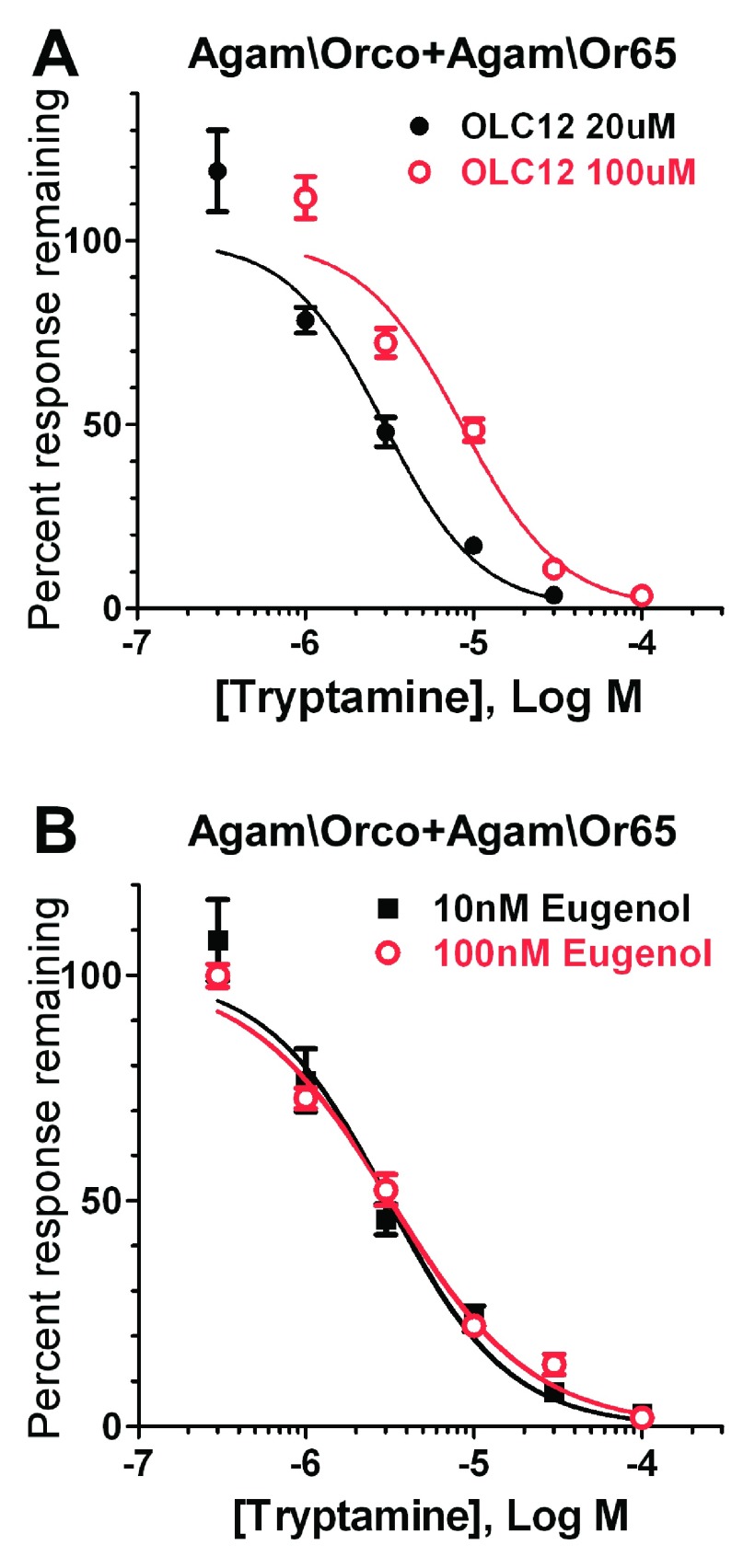
Tryptamine antagonism of odorant activation of an Agam\OR is non-competitive. **A**) Tryptamine competitively inhibits OLC12 activation of Agam\Orco+Agam\Or65. Altering the concentration of Orco agonist (OLC12) shifts the tryptamine inhibition curve. The IC _50_ for tryptamine inhibition of Agam\Orco+Agam\Or65 activation by 20µM OLC12 (2.9 ± 0.5µM, n = 3) is significantly different (p<0.0001, F-test) from the IC _50_ for tryptamine inhibition of Agam\Orco+Agam\Or65 activation by 100µM OLC12 (8.5 ± 1.1µM, n = 3). **B**) Tryptamine non-competitively inhibits odorant activation of Agam\Orco+Agam\Or65. Altering odorant (eugenol) concentration fails to shift the tryptamine inhibition curve. The IC _50_ values for tryptamine inhibition of responses to 10nM eugenol (3.1 ± 0.4µM, n = 4), and 100nM eugenol (3.2 ± 0.3µM, n = 3) did not differ (p=0.7172, F-test).

To screen for agonist activity, oocytes were exposed to 30 sec applications of candidate compounds with 5 min washes between applications ( Figure 2A). For the concentration-response protocol ( Table 1), applications were for 20 sec at a flow rate of 1.65 ml/min. To measure antagonist activity at Orco ( Figure 2B, 2C, Figure 3, Figure 4A and Figure 5A), oocytes were exposed to two 60 sec applications of the synthetic Orco agonist OLC12 (2-((4-Ethyl-5-(4-pyridinyl)-4H-1,2,4-triazol-3-yl)sulfanyl)-N-(4-isopropylphenyl)acetamide) with 4 min washes between applications. Oocytes were then exposed to a 90 sec application of antagonist candidate, immediately followed by a 60 sec co-application of antagonist candidate and OLC12. The current response in the presence of antagonist candidate was compared to the mean of the preceding two responses to OLC12 alone and is presented as a percentage.

**Table 1.** Odorant and Orco agonist concentration-response curve values for Orco homomers and heteromeric ORs from several insect species. Concentration-response data was fit as described in Methods. n _H_ is the apparent Hill coefficient. Values are presented as mean ± SEM (n = 3-14).

Receptor	**Ligand** **(Normalizing Conc.)**	**EC _50_** **µM**	n _H_	Fit Max
Agam\Orco	OLC12 (30µM)	124 ± 9	2.4 ± 0.3	47 ± 2
Agam\Orco + Agam\Or31	Geranyl Acetate (30µM)	65 ± 23	1.0 ± 0.3	2.9 ± 0.3
Agam\Orco + Agam\Or65	Eugenol (1µM)	0.08 ± 0.01	0.9 ± 0.1	1.0 ± 0.03
Agam\Orco + Agam\Or65	OLC12 (30µM)	67 ± 6	2.0 ± 0.3	5.7 ± 0.3
Cqui\Orco	OLC12 (30µM)	95 ± 6	2.5 ± 0.3	48 ± 2
Dmel\Orco	OLC12 (10µM)	36 ± 4	3.9 ± 1.9	36 ± 4
Dmel\Orco + Dmel\Or35a	OLC12 (10µM)	20 ± 5	1.9 ± 0.8	4.7 ± 0.4
Onub\Orco + Onub\Or6	OLC12 (100µM)	100 ± 4	2.1 ± 0.2	2.0 ± 0.1

**Figure 6.**
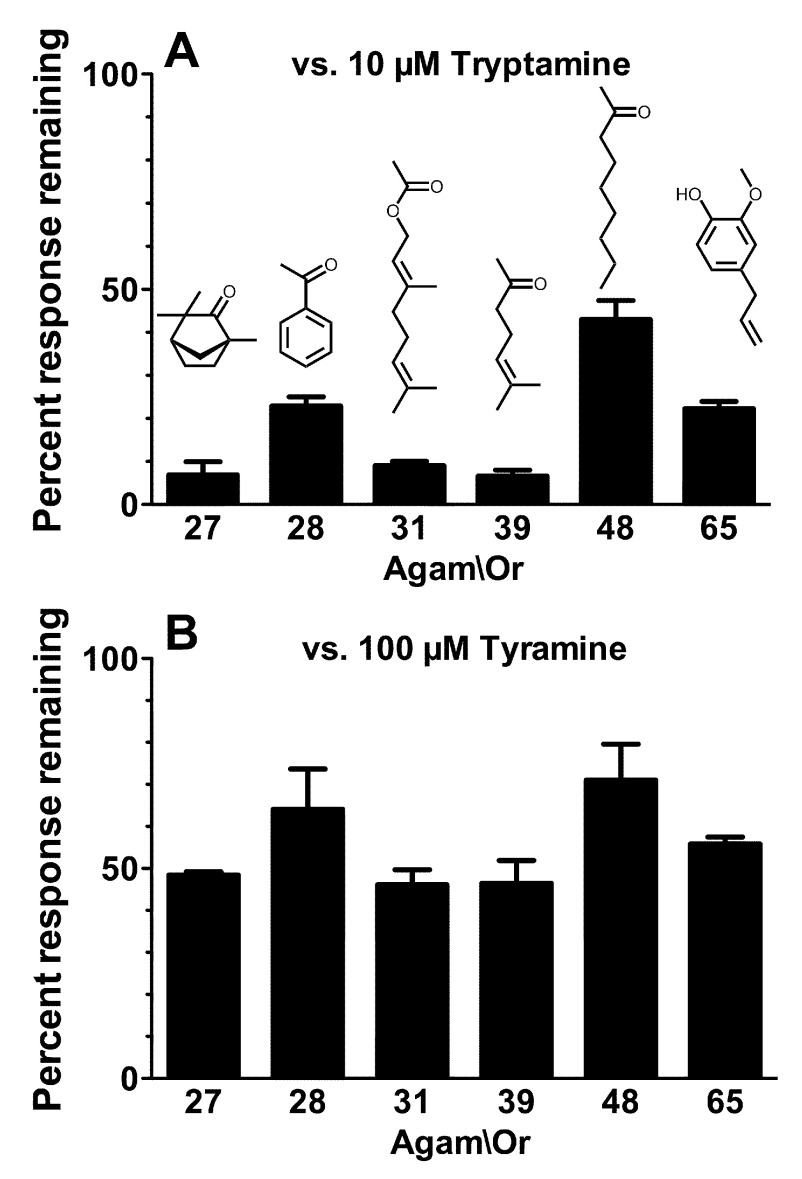
Tryptamine and tyramine inhibit odorant activation of multiple Agam\ORs. Current responses of oocytes expressing Agam\Orco+Agam\Or27 (activated by 3µM L-fenchone), Agam\Orco+Agam\Or28 (activated by 40µM acetophenone), Agam\Orco+Agam\Or31 (activated by 70µM geranyl acetate), Agam\Orco+Agam\Or39 (activated by 10µM 6-methyl-5-hepten-2-one), Agam\Orco+Agam\Or48 (activated by 3µM 2-nonanone), or Agam\Orco+Agam\Or65 (activated by 100nM eugenol) in the presence of 10µM tryptamine ( **A**) or 100µM tyramine ( **B**) were compared to the preceding response to odorant alone and are presented as mean ± SEM (n = 3). Odorant structures are shown.

To measure inhibition of odorant activation of heteromeric ORs ( Figures 4B, 4C, Figure 5B and Figure 6), oocytes were exposed to a 30 sec application of odorant followed by a 10 min wash. Oocytes were then exposed to a 90 sec application of tryptamine or tyramine, immediately followed by a 30 sec co-application of tryptamine or tyramine and odorant. The current response in the presence of antagonist candidate was compared to the preceding response to odorant alone and expressed as a percentage. In our previous work, we found that repeated odorant applications to some ORs could cause a progressive decrease in response amplitude ^31, 33^. For this reason, we then re-normalized antagonism data to the value obtained when the assay was run in the absence of antagonist candidate (sham). In the “sham” assay, oocytes were exposed to a 30 sec application of odorant followed by a 10 min wash and then exposed to a 90 sec application of ND96 (no antagonist candidate), immediately followed by a 30 sec application of odorant. The second odorant response was compared to the first response and expressed as a percentage. In Figure 4B, 4C, Figure 5B and Figure 6, the sham value for 100nM eugenol was 57 ± 3% (mean ± SEM, n = 3). In Figure 5B, the sham value for 10nM eugenol was 93 ± 4% (n = 4). In Figure 4B and C, the sham value for 1µM Z11-14:OAc was 82 ± 6% (n = 6) and the sham value for 150µM 2-phenylethanol was 92 ± 2% (n = 3). In Figure 6, the sham value for 3µM l-fenchone was 83 ± 1% (n = 3), the sham value for 40µM acetophenone was 92 ± 1% (n = 3), the sham value for 70µM geranyl acetate was 97 ± 1% (n = 3), the sham value for 10µM 6-methyl-5-hepten-2-one was 94 ± 2% (n = 3) and the sham value for 3µM 2-nonanone was 81 ± 1% (n = 3).

Initial analysis of electrophysiological data was done using Clampfit 9.1 software (Molecular Devices). Curve fitting and statistical analyses were done using Prism 5 (Graphpad). Concentration-inhibition data were fit to the equation: I = I _max_/(1+ (X/IC _50_) ^n^) where I represents the current response at a given concentration of inhibitor, X; I _max_ is the maximal response in the absence of inhibitor; IC _50_ is the concentration of inhibitor present that still allows a half maximal response from odorant; n is the apparent Hill coefficient. Concentration-response data were fit to the equation: I = I _max_/(1+(EC _50_/X) ^n^) where I represents the current response at a given concentration of odorant, X; I _max_ is the maximal response; EC _50_ is the concentration of agonist yielding a half maximal response; n is the apparent Hill coefficient. Statistical significance (p<0.05) was assessed using a two-tailed unpaired *t* test, an F test, or a one-way analysis of variance followed by the Dunnett's post-test, as appropriate.

## Results

Inhibition of odorant and Orco agonist initiated current responses of oocytes expressing insect odorant receptors by various amines Figure 2C Data. Current responses of oocytes expressing Cqui\Orco to 30µM OLC12 (~EC5) in the presence of 100µM of each compound presented as a percentage of the average of two preceding responses to OLC12 alone. Sham is an application of ND96 (no antagonist candidate). Figure 3A Data. Current responses of oocytes expressing Cqui\Orco to 30µM OLC12 (~EC5) in the presence of various concentrations of tryptamine, tyramine or phenethylamine are presented as a percentage of the response to OLC12 alone.
Figure 3B Data_30µM OLC12 curve. Current responses of oocytes expressing Cqui\Orco to 30µM OLC12 in the presence of various concentrations of tryptamine are presented as a percentage of the response to OLC12 alone.
Figure 3B Data_100µM OLC12 curve. Current responses of oocytes expressing Cqui\Orco to 100µM OLC12 in the presence of various concentrations of tryptamine are presented as a percentage of the response to OLC12 alone.
Figure 4A Data. Oocytes expressing Cqui\Orco, Agam\Orco or Dmel\Orco were activated by OLC12 (Cqui\Orco, 30µM; Agam\Orco, 30µM; Dmel\Orco, 20µM). Values are the current responses in the presence of 10µM tryptamine presented as a percentage of the average of two preceding responses to OLC12 alone.
Figure 4B Data. Oocytes expressing Agam\Orco+Agam\Or65 were activated by 100nM eugenol, oocytes expressing Onub\Orco+Onub\Or6 were activated by 1µM Z11-14:OAc, oocytes expressing Mcar\Orco+Mcar\Or5 were activated by 150µM 2-phenylethanol. Values represent the normalized (see Methods) percentage of current responses in the presence of 10µM tryptamine to the preceding response to odorant alone.
Figure 4C Data. Oocytes expressing Agam\Orco+Agam\Or65 were activated by 100nM eugenol, oocytes expressing Onub\Orco+Onub\Or6 were activated by 1µM Z11-14:OAc, oocytes expressing Mcar\Orco+Mcar\Or5 were activated by 150µM 2-phenylethanol. Values represent the normalized (see Methods) percentage of current responses in the presence of 100µM tyramine to the preceding response to odorant alone.
Figure 5A Data_OLC12 20µM curve. Current responses of oocytes expressing Agam\Orco+Agam\Or65 to 20µM OLC12 in the presence of various concentrations of tryptamine are presented as a percentage of the response to OLC12 alone.
Figure 5A Data_OLC12 100µM curve. Current responses of oocytes expressing Agam\Orco+Agam\Or65 to 100µM OLC12 in the presence of various concentrations of tryptamine are presented as a percentage of the response to OLC12 alone.
Figure 5B Data_Eugenol 10nM curve. Oocytes expressing Agam\Orco+Agam\Or65 were activated by 10nM eugenol. Values are the normalized (see Methods) percentage of current responses in the presence of various concentrations of tryptamine compared to the preceding response to odorant alone.
Figure 5B Data_Eugenol 100nM curve. Oocytes expressing Agam\Orco+Agam\Or65 were activated by 100nM eugenol. Values are the normalized (see Methods) percentage of current responses in the presence of various concentrations of tryptamine compared to the preceding response to odorant alone.
Figure 6A Data. Values represent the normalized (see Methods) percentage of current responses of oocytes expressing Agam\Orco+Agam\Or27 (activated by 3µM L-fenchone), Agam\Orco+Agam\Or28 (activated by 40µM acetophenone), Agam\Orco+Agam\Or31 (activated by 70µM geranyl acetate), Agam\Orco+Agam\Or39 (activated by 10µM 6-methyl-5-hepten-2-one), Agam\Orco+Agam\Or48 (activated by 3µM 2-nonanone), or Agam\Orco+Agam\Or65 (activated by 100nM eugenol) in the presence of 10µM tryptamine, as compared to the preceding response to odorant alone.
Figure 6B Data. Values represent the normalized (see Methods) percentage of current responses of oocytes expressing Agam\Orco+Agam\Or27 (activated by 3µM L-fenchone), Agam\Orco+Agam\Or28 (activated by 40µM acetophenone), Agam\Orco+Agam\Or31 (activated by 70µM geranyl acetate), Agam\Orco+Agam\Or39 (activated by 10µM 6-methyl-5-hepten-2-one), Agam\Orco+Agam\Or48 (activated by 3µM 2-nonanone), or Agam\Orco+Agam\Or65 (activated by 100nM eugenol) in the presence of 10µM tyramine, as compared to the preceding response to odorant alone.

To screen a panel of biogenic and trace amines ( Figure 1), we expressed Orco from *Culex quinquefasciatus* (Southern House Mosquito) in *Xenopus* oocytes and recorded ligand-induced current responses using two-electrode voltage clamp electrophysiology (see Methods). Orco subunits from several species, including Cqui\Orco, have been shown to form homomeric channels when heterologously expressed in the absence of odorant-binding subunits ^27, 33^. This convenient property of Orco allowed us to perform the initial screen without potentially confounding interactions with odorant-binding subunits. Successful functional expression of Cqui\Orco was confirmed by application of OLC12, a previously identified Orco specific agonist ^31^. While OLC12 elicited robust current responses, none of the amines displayed agonist activity at Cqui\Orco ( Figure 2A). Next we screened the amines for antagonist activity by applying 30µM OLC12 (∼EC _5_) to activate Cqui\Orco and co-applying 100µM of each amine ( Figure 2B, C). Several amines were able to inhibit OLC12 activation of Cqui\Orco. Tryptamine was the most effective antagonist, blocking more than 90% of the OLC12 response (92 ± 2% inhibition). Highly significant inhibition (p<0.001) was also observed for phenethylamine (41 ± 1%), tyramine (40 ± 5%), gramine (30 ± 4%) and serotonin 23 ± 3%), but the extent of inhibition was less than 50%, suggesting relatively low affinity interactions. Histamine (16 ± 8%), melatonin (13 ± 1%) and epinephrine (9 ± 3%) also displayed significant (p<0.01), but modest, inhibition of the OLC12 current. Octopamine, dopamine and norepinephrine were inactive in this assay.

In Figure 3A, we constructed concentration-inhibition curves for block of Cqui\Orco activity in order to quantitatively evaluate the inhibitory potency of tryptamine, as well as phenethylamine and tyramine, representing the less effective amines. Tryptamine was clearly the most potent of these antagonists, inhibiting Cqui\Orco with an IC _50_ of 4.7 ± 0.7µM, a value similar to that of the most potent synthetic Orco antagonists that we identified in our previous work ^33^. Phenethylamine (IC _50_ = 117 ± 12µM) and tyramine (IC _50_ = 157 ± 22µM) were substantially less potent than tryptamine (25-fold and 33-fold, respectively). Previously identified Orco antagonists inhibited OLC12 activation of Orco through a competitive mechanism ^31, 33^. To determine whether tryptamine was also a competitive antagonist of Orco, we measured blockade of Cqui\Orco achieved by tryptamine when the OLC12 concentration was increased from 30µM to 100µM ( Figure 3B). Tryptamine was significantly less effective at inhibiting responses to 100µM OLC12 (IC _50_ = 143 ± 18µM, p<0.0001, F-test), indicating that tryptamine is a competitive antagonist of Cqui\Orco.

We next asked whether tryptamine could also inhibit Orco from other insect species. In addition to Cqui\Orco, we tested Agam\Orco from *An. gambiae* (human malaria vector mosquito) and Dmel\Orco from *D. melanogaster*. Co-application of 10µM tryptamine inhibited OLC12 activation of Orco from each of these three insect species ( Figure 4A). We then wondered whether tryptamine could also inhibit odorant activation of heteromeric insect ORs containing both Orco and odorant binding subunits. We chose ORs from three insect orders: Agam\Orco+Agam\Or65 from *An. gambiae* (Order Diptera) that responds to the eugenol ^25^; Onub\Orco+Onub\Or6 from *O. nubilalis* (European Corn Borer, Order Lepidoptera) that responds to the pheromone Z11-14:OAc ^45^; and Mcar\Orco+Mcar\Or5 from *M. caryae* (Long-Horned Beetle, Order Coleoptera) that responds to 2-phenylethanol ^44^. We chose to proceed with an OR from *An. gambiae* instead of *Cx. quinquefasciatus* for two reasons. The best characterized of the Cqui\Or subunits respond to indoles ^23, 24^, which are structurally related to tryptamine and might confound our experiments. Also, the Agam\Or subunit family has been more extensively characterized ^10, 25^, offering more options for OR expression (see below). Each odorant was applied at or near the EC _50_ concentration ( Table 1, ^44, 45^). Co-application of 10µM tryptamine resulted in substantial inhibition of each receptor ( Figure 4B). We also examined tyramine. While tyramine is a low potency Orco antagonist ( Figure 3A), it is a major neurotransmitter in insects ^37^. Tyramine was also able to reduce odorant activation of these ORs, but was less effective than tryptamine ( Figure 4C). These results suggest that tryptamine and tyramine are broadly active antagonists of insect ORs.

Several previously identified Orco antagonists have been shown to inhibit odorant activation of insect ORs through a non-competitive mechanism ^31– 33^. To determine whether the tryptamine inhibition of odorant activation that we observed in Figure 4 was also non-competitive, we examined the effect of tryptamine on activation of the heteromeric Agam\Orco+Agam\Or65 in more detail ( Figure 5). When the concentration of Orco directed agonist (OLC12) was increased, the tryptamine inhibition curve was significantly shifted to the right ( Figure 5A). However, when the concentration of odorant agonist (eugenol) was increased, the tryptamine inhibition curve did not shift ( Figure 5B). These results indicate that, similar to previously identified synthetic Orco antagonist compounds, tryptamine is a competitive antagonist of direct activation of Orco and a non-competitive antagonist of odorant activation of the OR.

The ability of tryptamine to interact with Orco and exert a non-competitive inhibitory effect on odorant activation of a heteromeric OR ( Figure 5) suggests that tryptamine should be able to inhibit activation of a variety of ORs activated by diverse odorants. To examine this possibility, we tested the ability of tryptamine to inhibit odorant activation of ORs formed by Agam\Orco and each of six different odorant-binding subunits chosen from across the *An. gambiae* OR gene family ^46^. We activated each OR with a previously identified cognate odorant ^25^ at a concentration at or near the EC _50_ ( Table 1, ^25^). In addition to Agam\Orco+Agam\Or65 (activated by eugenol), we tested Agam\Orco+Agam\Or27 (activated by L-fenchone), Agam\Orco+Agam\Or28 (activated acetophenone), Agam\Orco+Agam\Or31 (activated by geranyl acetate), Agam\Orco+Agam\Or39 (activated by 6-methyl-5-hepten-2-one) and Agam\Orco+Agam\Or48 (activated by 2-nonanone). With the exception of Agam\Or39 and Agam\Or48, which display overlapping odorant specificities at 4 odorants, there is little or no similarity among the odorant specificities of these six odorant-binding subunits ^25^. In each case, 10µM tryptamine was able to inhibit odorant activation of the receptor, despite the disparate odorant-binding subunits and diverse odorant structures ( Figure 6). Tyramine was also able to inhibit odorant activation of each of these receptors, but was less effective than tryptamine (note that tyramine is applied at 100µM). We conclude that tryptamine and tyramine are general antagonists of insect ORs.

## Discussion

Animals use a variety of biogenic and trace amines as neurotransmitters and neuromodulators. These include compounds derived from tyrosine (dopamine, norepinephrine, epinephrine, tyramine, octopamine and phenethylamine), tryptophan (serotonin, melatonin and tryptamine) and histidine (histamine) ^47^. Dopamine and serotonin play a variety of roles in the insect nervous system ^48– 50^. In addition, insects use octopamine, histamine and tyramine as neurotransmitters ^48– 53^. Melatonin also appears to exert neuromodulatory effects in insects ^54, 55^. Interestingly, many of these amines modulate the olfactory system ^56– 58^.

Recent reports ^27, 32^, together with our previous findings ^31, 33^, have revealed the existence of a ligand-binding site on the Orco subunit and that inhibition of odorant activation through a non-competitive mechanism may be a general property of Orco-directed antagonists. Our current results suggest that endogenous and exogenous natural compounds serve as Orco ligands and modulate insect olfaction. While tyramine is a major neurotransmitter in insects ^53^, its low potency in our assay ( Figure 3) suggests that it might not serve as an endogenous OR modulator. However, the function of an endogenous Orco antagonist is unlikely to be the complete block of OR function. Rather, an endogenous Orco antagonist might be used to diminish olfactory sensitivity by inhibiting a fraction of the available receptors. For tyramine, such inhibition could occur at concentrations ranging from 10µM to 30µM. Alternatively, there may be additional, more potent, but as yet uncharacterized, endogenous Orco antagonists that can decrease olfactory sensitivity at lower concentrations.

In contrast to the low potency of tyramine, we found tryptamine to be a high potency Orco antagonist. Tryptamine inhibited odorant activation of an OR with an IC _50_ in the low micromolar range ( Figure 5). While it is currently unclear whether tryptamine is endogenous to insects, tryptamine and similar compounds, such as gramine, are produced by a variety of plants and are thought to serve as a defense against insect herbivores ^42, 59^. Various tryptamine analogs have been proposed as larvicides ^60^ and when tryptamine is caused to accumulate in poplar and tobacco, through ectopic expression of tryptophan decarboxylase, the feeding behavior of insects that target these plants is altered ^41^. Tryptamine-based structures also act on various receptors and transporters, particularly those involved in serotonergic neurotransmission, exerting psychedelic effects in humans. Indeed, many plant derived and synthetic hallucinogens are based on the tryptamine and phenethylamine scaffolds ^61– 63^. Interestingly, the potency that we observed for tryptamine inhibition of odorant activation of an insect OR ( Figure 5) is similar to the potency for tryptamine inhibition of the *D. melanogaster* serotonin transporter ^64^.

Might there also be natural endogenous or exogenous Orco agonists? An endogenous Orco agonist could serve to increase olfactory sensitivity, perhaps in a circadian fashion, to alter behavior during critical foraging or mating periods. An exogenous, plant-derived Orco agonist would, by activating all ORs through Orco, serve as an olfactory “confusant” and might alter the feeding behavior of insect herbivores. The limited screen of 11 compounds that we conducted here did not identify any Orco agonists, but more extensive screening is clearly warranted.

Several synthetic Orco antagonists have been shown to inhibit odorant activation of ORs through an allosteric mechanism ^31– 33^. The ability of these compounds to inhibit multiple ORs from a variety of species is likely due to the high conservation of Orco across the insects ^12^. Similarly, we found that tryptamine and tyramine, acting as Orco antagonists, could inhibit odorant activation of ORs from insect species chosen from three different orders: Diptera ( *An. gambiae*), Lepidoptera ( *O. nubilalis*) and Coleoptera ( *M. caryae*). Furthermore, when we examined multiple ORs from a single species ( *An. gambiae*), we found that tryptamine and tyramine blocked odorant activation of each receptor. The action of these compounds through Orco allowed blockade to occur despite the highly diverse odorant-binding subunits used to form the receptors and the different odorant structures used to activate the receptors. Interestingly, while all six receptors were inhibited, the extent of inhibition varied depending on the odorant-binding subunit present and the pattern of variation was similar for tryptamine and tyramine. This suggests differences in allosteric coupling between Orco and the various odorant-binding subunits. Also, while we showed that tryptamine is a potent inhibitor of odorant activation of Agam\Or65+Agam\Orco, the results we present in Figure 6 suggest that tryptamine is even more potent at other ORs, such as those formed by Agam\Or27, Agam\Or31 and Agam\Or39. Our current results with naturally occurring amines, together with previous reports with synthetic compounds ^27, 31– 33, 35^ strongly suggest that: 1) allosteric antagonism of odorant activation of ORs is a general property of Orco antagonists; 2) Orco antagonists are broadly active at ORs of many insect species; and 3) Orco is an important target for the development of novel insect repellants. The broad activity of Orco directed compounds across many insect species that has been observed to date suggests that these compounds may have limited agricultural utility, since both pests and pollinators could be affected. Determining whether species-specific Orco ligands can be developed will require further effort. What is clear, however, is that the pursuit of new, synthetic Orco directed ligands (both agonists and antagonists) is a promising direction for the development of new, more effective insect repellants that can aid in controlling the spread of insect-borne diseases.

## Data availability

figshare: Inhibition of odorant and Orco agonist initiated current responses of oocytes expressing insect odorant receptors by various amines, doi: 10.6084/m9.figshare.977791 ^65^

## References

[1] Klein AM Vaissière BE Cane JH: Importance of pollinators in changing landscapes for world crops. *Proc Biol Sci.* 2007;274(1608):303–313 10.1098/rspb.2006.3721 17164193 PMC1702377

[2] Kremen C Williams NM Aizen MA: Pollination and other ecosystem services produced by mobile organisms: a conceptual framework for the effects of land-use change. *Ecol Lett.* 2007;10(4):299–314 10.1111/j.1461-0248.2007.01018.x 17355569

[3] Abate T Ampofo JK: Insect pests of beans in Africa: their ecology and management. *Annu Rev Entomol.* 1996;41:45–73 10.1146/annurev.en.41.010196.000401 15012324

[4] Bardner R Fletcher KE: Insect Infestations and Their Effects on Growth and Yield of Field Crops: a Review. *Bull Entomol Res.* 1974;64(1):141–160 10.1017/S0007485300027061

[5] Gahukar RT: Insect Pests of Millets and Their Management: a Review. *Trop Pest Manage.* 35(4):382–391 10.1080/09670878909371411

[6] Gratz NG: Critical review of the vector status of Aedes albopictus. *Med Vet Entomol.* 2004;18(3):215–227 10.1111/j.0269-283X.2004.00513.x 15347388

[7] Sinka ME Rubio-Palis Y Manguin S: The dominant Anopheles vectors of human malaria in the Americas: occurrence data, distribution maps and bionomic precis. *Parasit Vectors.* 2010;3:72 10.1186/1756-3305-3-72 20712879 PMC2936890

[8] Pages F Faulde M Orlandi-Pradines E: The past and present threat of vector-borne diseases in deployed troops. *Clin Microbiol Infect.* 2010;16(3):209–224 10.1111/j.1469-0691.2009.03132.x 20222896

[9] Hallem EA Carlson JR: Coding of odors by a receptor repertoire. *Cell.* 2006;125(1):143–160 10.1016/j.cell.2006.01.050 16615896

[10] Carey AF Wang G Su CY: Odorant reception in the malaria mosquito Anopheles gambiae. *Nature.* 2010;464(7285):66–71 10.1038/nature08834 20130575 PMC2833235

[11] Vosshall LB Amrein H Morozov PS: A spatial map of olfactory receptor expression in the Drosophila antenna. *Cell.* 1999;96(5):725–736 10.1016/S0092-8674(00)80582-6 10089887

[12] Larsson MC Domingos AI Jones WD: Or83b encodes a broadly expressed odorant receptor essential for Drosophila olfaction. *Neuron.* 2004;43(5):703–714 10.1016/j.neuron.2004.08.019 15339651

[13] Nakagawa T Vosshall LB: Controversy and consensus: noncanonical signaling mechanisms in the insect olfactory system. *Curr Opin Neurobiol.* 2009;19(3):284–292 10.1016/j.conb.2009.07.015 19660933 PMC2752668

[14] Neuhaus EM Gisselmann G Zhang W: Odorant receptor heterodimerization in the olfactory system of Drosophila melanogaster. *Nat Neurosci.* 2005;8(1):15–17 10.1038/nn1371 15592462

[15] Benton R Sachse S Michnick SW: Atypical membrane topology and heteromeric function of Drosophila odorant receptors *in vivo*. *PLoS Biol.* 2006;4(2):e20 10.1371/journal.pbio.0040020 16402857 PMC1334387

[16] Laissue PP Vosshall LB: The olfactory sensory map in Drosophila. *Adv Exp Med Biol.* 2008;628:102–114 10.1007/978-0-387-78261-4_7 18683641

[17] Goldman AL Van der Goes van Naters W Lessing D: Coexpression of two functional odor receptors in one neuron. *Neuron.* 2005;45(5):661–666 10.1016/j.neuron.2005.01.025 15748842

[18] Wicher D Schäfer R Bauernfeind R: Drosophila odorant receptors are both ligand-gated and cyclic-nucleotide-activated cation channels. *Nature.* 2008;452(7190):1007–1011 10.1038/nature06861 18408711

[19] Sato K Pellegrino M Nakagawa T: Insect olfactory receptors are heteromeric ligand-gated ion channels. *Nature.* 2008;452(7190):1002–1006 10.1038/nature06850 18408712

[20] Nichols AS Luetje CW: Transmembrane segment 3 of Drosophila melanogaster odorant receptor subunit 85b contributes to ligand-receptor interactions. *J Biol Chem.* 2010;285(16):11854–11862 10.1074/jbc.M109.058321 20147286 PMC2852922

[21] Nichols AS Chen S Luetje CW: Subunit contributions to insect olfactory receptor function: channel block and odorant recognition. *Chem Senses.* 2011;36(9):781–790 10.1093/chemse/bjr053 21677030 PMC3195787

[22] Pask GM Jones PL Rutzler M: Heteromeric Anopheline odorant receptors exhibit distinct channel properties. *PLoS One.* 2011;6(12):e28774 10.1371/journal.pone.0028774 22174894 PMC3235152

[23] Hughes DT Pelletier J Luetje CW: Odorant receptor from the southern house mosquito narrowly tuned to the oviposition attractant skatole. *J Chem Ecol.* 2010;36(8):797–800 10.1007/s10886-010-9828-9 20623327 PMC2908433

[24] Pelletier J Hughes DT Luetje CW: An odorant receptor from the southern house mosquito Culex pipiens quinquefasciatus sensitive to oviposition attractants. *PLoS One.* 2010;5(4):e10090 10.1371/journal.pone.0010090 20386699 PMC2851645

[25] Wang G Carey AF Carlson JR: Molecular basis of odor coding in the malaria vector mosquito Anopheles gambiae. *Proc Natl Acad Sci U S A.* 2010;107(9):4418–4423 10.1073/pnas.0913392107 20160092 PMC2840125

[26] Xia Y Wang G Buscariollo D: The molecular and cellular basis of olfactory-driven behavior in Anopheles gambiae larvae. *Proc Natl Acad Sci U S A.* 2008;105(17):6433–6438 10.1073/pnas.0801007105 18427108 PMC2359781

[27] Jones PL Pask GM Rinker DC: Functional agonism of insect odorant receptor ion channels. *Proc Natl Acad Sci U S A.* 2011;108(21):8821–8825 10.1073/pnas.1102425108 21555561 PMC3102409

[28] Rinker DC Jones PL Jason pitts R: Novel high-throughput screens of Anopheles gambiae odorant receptors reveal candidate behavior-modifying chemicals for mosquitoes. *Physiol Entomol.* 2012;37:33–41 10.1111/j.1365-3032.2011.00821.x PMC7123412 32255891

[29] Carey AF Carlson JR: Insect olfaction from model systems to disease control. *Proc Natl Acad Sci U S A.* 2011;108(32):12987–12995 10.1073/pnas.1103472108 21746926 PMC3156210

[30] Ramdya P Benton R: Evolving olfactory systems on the fly. *Trends Genet.* 2010;26(7):307–316 10.1016/j.tig.2010.04.004 20537755

[31] Chen S Luetje CW: Identification of new agonists and antagonists of the insect odorant receptor co-receptor subunit. *PLoS One.* 2012;7(5):e36784 10.1371/journal.pone.0036784 22590607 PMC3348135

[32] Jones PL Pask GM Romaine IM: Allosteric antagonism of insect odorant receptor ion channels. *PLoS One.* 2012;7(1):e30304 10.1371/journal.pone.0030304 22272331 PMC3260273

[33] Chen S Luetje CW: Phenylthiophenecarboxamide antagonists of the olfactory receptor co-receptor subunit from a mosquito. *PLoS One.* 2013;8(12):e84575 10.1371/journal.pone.0084575 24358366 PMC3866151

[34] Jones WD Nguyen TA Kloss B: Functional conservation of an insect odorant receptor gene across 250 million years of evolution. *Curr Biol.* 2005;15(4):R119–121 10.1016/j.cub.2005.02.007 15723778

[35] Bohbot JD Dickens JC: Odorant receptor modulation: ternary paradigm for mode of action of insect repellents. *Neuropharmacology.* 2012;62(5-6):2086–2095 10.1016/j.neuropharm.2012.01.004 22269900

[36] Blenau W Thamm M: Distribution of serotonin (5-HT) and its receptors in the insect brain with focus on the mushroom bodies: lessons from Drosophila melanogaster and Apis mellifera. *Arthropod Struct Dev.* 2011;40(5):381–394 10.1016/j.asd.2011.01.004 21272662

[37] Cazzamali G Klaerke DA Grimmelikhuijzen CJ: A new family of insect tyramine receptors. *Biochem Biophys Res Commun.* 2005;338(2):1189–1196 10.1016/j.bbrc.2005.10.058 16274665

[38] Nassel DR: Histamine in the brain of insects: a review. *Microsc Res Tech.* 1999;44(2-3):121–136 10.1002/(SICI)1097-0029(19990115/01)44:2/3<121::AID-JEMT6>3.0.CO;2-F 10084821

[39] Roeder T: Octopamine in invertebrates. *Prog Neurobiol.* 1999;59(5):533–561 10.1016/S0301-0082(99)00016-7 10515667

[40] Cai QN Han Y Cao YZ: Detoxification of gramine by the cereal aphid Sitobion avenae. *J Chem Ecol.* 2009;35(3):320–325 10.1007/s10886-009-9603-y 19224277

[41] Gill RI Ellis BE Isman MB: Tryptamine-induced resistance in tryptophan decarboxylase transgenic poplar and tobacco plants against their specific herbivores. *J Chem Ecol.* 2003;29(4):779–793 10.1023/A:1022983529555 12775143

[42] Thomas JC Saleh EF Alammar N: The indole alkaloid tryptamine impairs reproduction in Drosophila melanogaster. *J Econ Entomol.* 1998;91(4):841–846 9725032 10.1093/jee/91.4.841

[43] Liman ER Tytgat J Hess P: Subunit stoichiometry of a mammalian K+ channel determined by construction of multimeric cDNAs. *Neuron.* 1992;9(5):861–871 10.1016/0896-6273(92)90239-A 1419000

[44] Mitchell RF Hughes DT Luetje CW: Sequencing and characterizing odorant receptors of the cerambycid beetle Megacyllene caryae. *Insect Biochem Mol Biol.* 2012;42(7):499–505 10.1016/j.ibmb.2012.03.007 22504490 PMC3361640

[45] Wanner KW Nichols AS Allen JE: Sex pheromone receptor specificity in the European corn borer moth, Ostrinia nubilalis. *PLoS One.* 2010;5(1):e8685 10.1371/journal.pone.0008685 20084285 PMC2801615

[46] Hill CA Fox AN Pitts RJ: G protein-coupled receptors in Anopheles gambiae. *Science.* 2002;298(5591):176–178 10.1126/science.1076196 12364795

[47] Zucchi R Chiellini G Scanlan TS: Trace amine-associated receptors and their ligands. *Br J Pharmacol.* 2006;149(8):967–978 10.1038/sj.bjp.0706948 17088868 PMC2014643

[48] Birmingham JT Tauck DL: Neuromodulation in invertebrate sensory systems: from biophysics to behavior. *J Exp Biol.* 2003;206(Pt 20):3541–3546 10.1242/jeb.00601 12966045

[49] Grosmaitre X Marion-Poll F Renou M: Biogenic amines modulate olfactory receptor neurons firing activity in Mamestra brassicae. *Chem Senses.* 2001;26(6):653–661 10.1093/chemse/26.6.653 11473931

[50] Perry CJ Barron AB: Neural mechanisms of reward in insects. *Annu Rev Entomol.* 2013;58:543–562 10.1146/annurev-ento-120811-153631 23020615

[51] Cole SH Carney GE McClung CA: Two functional but noncomplementing Drosophila tyrosine decarboxylase genes: distinct roles for neural tyramine and octopamine in female fertility. *J Biol Chem.* 2005;280(15):14948–14955 10.1074/jbc.M414197200 15691831

[52] Monastirioti M: Biogenic amine systems in the fruit fly Drosophila melanogaster. *Microsc Res Tech.* 1999;45(2):106–121 10.1002/(SICI)1097-0029(19990415)45:2<106::AID-JEMT5>3.0.CO;2-3 10332728

[53] Roeder T: Tyramine and octopamine: ruling behavior and metabolism. *Annu Rev Entomol.* 2005;50:447–477 10.1146/annurev.ento.50.071803.130404 15355245

[54] Bembenek J Sehadova H Ichihara N: Day/night fluctuations in melatonin content, arylalkylamine N-acetyltransferase activity and NAT mRNA expression in the CNS peripheral tissues and hemolymph of the cockroach, Periplaneta americana. *Comp Biochem Physiol B Biochem Mol Biol.* 2005;140(1):27–36 10.1016/j.cbpc.2004.03.017 15621506

[55] Richter K Peschke E Peschke D: Effect of melatonin on the release of prothoracicotropic hormone in the brain of Periplaneta americana (Blottodea: Blattidae). *Eur J Entomol.* 1999;96:341–345 Reference Source

[56] Dacks AM Riffell JA Martin JP: Olfactory modulation by dopamine in the context of aversive learning. *J Neurophysiol.* 2012;108(2):539–550 10.1152/jn.00159.2012 22552185 PMC3404788

[57] Huser A Rohwedder A Apostolopoulou AA: The serotonergic central nervous system of the Drosophila larva: anatomy and behavioral function. *PLoS One.* 2012;7(10):e47518 10.1371/journal.pone.0047518 23082175 PMC3474743

[58] Zhukovskaya MI: Modulation by octopamine of olfactory responses to nonpheromone odorants in the cockroach, Periplaneta americana L. *Chem Senses.* 2012;37(5):421–429 10.1093/chemse/bjr121 22281532

[59] Sun XQ Zhang MX Yu JY: Glutathione S-transferase of brown planthoppers (Nilaparvata lugens) is essential for their adaptation to gramine-containing host plants. *PLoS One.* 2013;8(5):e64026 10.1371/journal.pone.0064026 23700450 PMC3659104

[60] Oliveira RR Brito TB Nepel A: Synthesis, Activity, and QSAR Studies of Tryptamine Derivatives on Third-instar Larvae of Aedes aegypti Linn. *Med Chem.* 2013 Epub ahead of print. 10.2174/1573406409666131202144010 24295020

[61] McKenna DJ: Plant hallucinogens: springboards for psychotherapeutic drug discovery. *Behav Brain Res.* 1996;73(1-2):109–116 8788486 10.1016/0166-4328(96)00079-4

[62] Shulgin A Shulgin A: Pihkal: A chemical love story. (Transform Press, Berkeley, California).1991;p 978 Reference Source

[63] Shulgin A Shulgin A: Tihkal: The continuation. (Transform Press, Berkeley, California).1997;p 804 Reference Source

[64] Adkins EM Barker EL Blakely RD: Interactions of tryptamine derivatives with serotonin transporter species variants implicate transmembrane domain I in substrate recognition. *Mol Pharmacol.* 2001;59(3):514–523 10.1124/mol.59.3.514 11179447

[65] Chen S Luetje CW: Inhibition of odorant and Orco agonist initiated current responses of oocytes expressing insect odorant receptors by various amines. *Figshare.* 2014 Data Source

