# Supplementary material for: Trace amines inhibit insect odorant receptor function through antagonism of the co-receptor subunit
Source: F1000Res. 2014 Apr 3;3:84. [Version 1] doi: 10.12688/f1000research.3825.1 (PMC4097363; doi:10.12688/f1000research.3825.1)
Supplement: Peer review history

# Peer review: Referee response for version 1

**DOI:** 10.5256/f1000research.4098.r4357

**Published:** 2014-05-06

**Authors:** Coral Warr

**Affiliations:** 1 School of Biological Sciences, Monash University, Clayton, Victoria, Australia

In this study the authors report the identification of new antagonists for the Orco protein, a co-receptor for the ligand binding Olfactory receptor (Or) family in insects. This is of interest as they suggest Orco antagonists might be a useful approach to modifying insect behaviour. The authors screened a panel of biogenic amines and found several that functioned as Orco antagonists, of which tryptamine, naturally produced in plants, was the most effective. They further showed that this activity was conserved against Orco from a number of insect species. The paper is well written, the data is solid and well presented, and is appropriately analysed and interpreted. The caveat to the use of currently identified Orco antagonists in biocontrol, namely that they appear to affect Orco in all insects and are not species-specific, is appropriately acknowledged. My one feedback comment is that the justification for screening just amines was not really clear, and I wondered if the authors had also in fact screened other types of compounds? If so it would be valuable to other researchers to also mention any screened compounds that did not have any effect on Orco function.

I have read this submission. I believe that I have an appropriate level of expertise to confirm that it is of an acceptable scientific standard.

# Peer review: Referee response for version 1

**DOI:** 10.5256/f1000research.4098.r4395

**Published:** 2014-04-07

**Authors:** Joseph Dickens

**Affiliations:** 1 Invasive Insect Biocontrol and Behaviour Laboratory, United States Department of Agriculture, Beltsville, MD, USA

This is a well-written paper that documents the discovery of modulatory effects of biogenic amines on the responses of odorant receptor assemblages (OR = odorant receptor + Orco = odorant receptor co-receptor) in insects. Both plant- and insect-produced amines are shown to antagonize responses of OR/Orco complexes to known agonists through interactions suggested to be with Orco. The potential role of the amines in regulating insect chemosensory behavior is suggested, and Orco is proposed as an important target for development of novel insect repellents.

I have read this submission. I believe that I have an appropriate level of expertise to confirm that it is of an acceptable scientific standard.
